# Identifying strategies to advance equitable implementation of co-occurring mental health and substance use disorder treatment in drug treatment courts: A study protocol

**DOI:** 10.1017/cts.2023.14

**Published:** 2023-02-07

**Authors:** Ayorkor Gaba, Ashleigh LoVette, Bailey Pridgen, Marquita Taylor, Eva Woodward, Milagros C. Rosal, Melissa Anderson, David Smelson

**Affiliations:** 1 Department of Psychiatry, University of Massachusetts Chan Medical School, Worcester, MA, USA; 2 School of Nursing, Johns Hopkins University, Baltimore, MD, USA; 3 School of Public Health, Yale University, New Haven, CT, USA; 4 VA Center for Mental Healthcare and Outcomes Research, North Little Rock, AR, USA; 5 Department of Psychiatry, University of Arkansas for Medical Sciences, Little Rock, AR, USA; 6 Department of Population and Quantitative Health Sciences, University of Massachusetts Chan Medical School, Worcester, MA, USA

**Keywords:** Health equity, mental health, substance use, criminal legal, co-occurring disorder, implementation, community-engaged

## Abstract

**Introduction:**

Behavioral health treatment disparities by race and ethnicity are well documented across the criminal legal system. Despite criminal legal settings such as drug treatment courts (DTCs) increasingly adopting evidence-based programs (EBPs) to improve care, there is a dearth of research identifying strategies to advance equitable implementation of EBPs and reduce racial/ethnic treatment disparities. This paper describes an innovative approach to identify community- and provider-generated strategies to support equitable implementation of an evidence-based co-occurring mental health and substance use disorder intervention, called Maintaining Independence and Sobriety through Systems Integration, Outreach and Networking-Criminal Justice (MISSION-CJ), in DTCs.

**Methods/design:**

Guided by the Health Equity Implementation Framework, qualitative interviews and surveys will assess factors facilitating and hindering equitable implementation of MISSION-CJ in DTCs among 30 Black/African American and/or Hispanic/Latino persons served and providers. Concept mapping with sixty Black/African American and/or Hispanic/Latino persons served and providers will gather community- and provider-generated strategies to address identified barriers. Finally, an advisory board will offer iterative feedback on the data to guide toolkit development and inform equitable implementation of MISSION-CJ within DTCs.

**Conclusions:**

The paper illustrates a protocol of a study based in community-engaged research and implementation science to understand multilevel drivers of racial/ethnic disparities in co-occurring disorder treatment and identify opportunities for intervention and improvements within criminal legal settings.

## Introduction

### Racial/Ethnic Health Disparities in Criminal Legal Settings

Due to structural racism and historical inequities, Black/African American and/or Hispanic/Latino adults are overrepresented in the criminal legal system in the USA. Despite recent decreasing incarceration rates, Black/African American people are incarcerated in state prisons five times the rate of White individuals [1] and are 3.6 times more likely to be incarcerated in jail than their White counterparts [2]. Hispanic individuals are incarcerated in state prisons 1.3 times the rate of White individuals [1]. The overrepresentation of Black and Brown people in the criminal legal system has direct and indirect implications and effects on racial/ethnic behavioral health and healthcare disparities in the USA.

Up to 70% of those involved in the criminal legal system have a mental health disorder (MHD), substance use disorder (SUD), or co-occurring MHD and SUD (COD) [3,4]. Individuals with criminal legal involvement and a COD experience worse psychosocial and health outcomes than those without a COD [5–7]. National data also show significant racial/ethnic treatment disparities within this population. A large study of male arrestees (*N* = 18,421) in jails in 10 US cities found that Black/African American arrestees with mental health and/or SUD were less likely to engage in care [8], and both Black/African American and Hispanic/Latino arrestees were less likely to benefit from SUD treatment in comparison with White arrestees [8]. Other studies have shown that, despite lower prevalence of behavioral health conditions, Black/African American and Hispanic/Latino adults in the criminal legal system with behavioral health conditions are less likely to be diagnosed and less likely to receive treatment [7]. Black/African American and Hispanic/Latino adults in the criminal legal system with behavioral health conditions report less engagement and satisfaction with their treatment than their White counterparts [9].

Despite lower prevalence of CODs among Black/African American and Hispanic/Latino people, multiple health, social, and structural issues interact within this population to create heightened risk for disparities. For example, Black/African American people with mental health conditions are more likely to be incarcerated than people of other races [10]. Poor standard of care for mental health treatment in carceral settings and carceral practices themselves worsen mental health and further exacerbate racial/ethnic behavioral health and healthcare disparities [11]. Given the overrepresentation of Black/African American and/or Hispanic/Latino adults within the criminal legal system and documented racial/ethnic disparities in behavioral healthcare, criminal legal settings are an important place to identify and address drivers of racial/ethnic health disparities.

To date, implementation-focused approaches to reduce or eliminate behavioral health disparities have not been used in these settings. This study provides a protocol and considerations for practitioners within criminal legal and behavioral health systems, and researchers to inform and promote the utilization of community-engaged approaches in the implementation of evidence-based programs (EBPs) within criminal legal settings. We will conduct a study in which we partner with persons-served, defined as persons being supported or treated for or in recovery from MHD, SUD, and/or COD, and providers/administrators, defined as persons providing or overseeing treatment, support, and services to those with MHD, SUD, and/or COD. Together, we will understand drivers of behavioral health treatment disparities and co-develop a health equity-informed implementation toolkit for an evidence-based behavioral health intervention, called Maintaining Independence and Sobriety through Systems Integration, Outreach and Networking-Criminal Justice (MISSION-CJ) in drug treatment courts (DTCs).

### Drug Treatment Courts

DTCs are alternatives to incarceration programs to address individuals’ behavioral health and legal issues in lieu of incarceration. These courts serve many adults with a COD and leverage legal sanctions in exchange for court mandated and monitored treatment. While DTCs have gained popularity, with over 1,700 adult DTCs nationally, treatment outcomes have been modest for individuals with a COD. For example, having a COD significantly increases the odds of serious DTC program failure (e.g., new offense) and poorer behavioral health outcomes [12].

Furthermore, racial/ethnic disparities exist within DTCs. Black/African American and Hispanic/ Latino individuals in DTC are less likely to graduate successfully relative to White men [13]. In terms of treatment, Hispanic/Latino participants are significantly less likely than White participants to be placed in residential treatment for similar patterns of drug use, and Black/African American participants are less likely to receive medications for opioid and alcohol use disorder in DTC settings [14], thus, suggesting inequitable implementation of behavioral health interventions within DTCs.

### Evidence-Based COD Intervention in DTCs

Evidence-based COD interventions, such as MISSION-CJ [15], have been embedded within DTCs to offer comprehensive support for individuals with COD. MISSION-CJ is a time-limited, multicomponent treatment intervention adapted from the original MISSION model [16]. MISSION-CJ follows five evidence-based practices to effectively engage people with COD in treatment and supports [15]: (1) Critical Time Intervention case management [17], which offers intensive in-community support that decreases in intensity as participants transition to community-based care; (2) Dual Recovery Therapy, composed of 13 structured group treatment sessions designed to simultaneously treat COD [18]; (3) Peer Support, including 11 recovery-oriented group sessions delivered by an individual with lived experience of COD and criminal legal involvement; (4) Vocational and educational support; and (5) Trauma-informed care. MISSION-CJ adds the Risk-Need-Responsivity framework, which utilizes assessment of recidivism risk to tailor treatment planning to address dynamic criminogenic needs driving offending behaviors and responsivity factors (e.g., gender, age, ethnicity, etc.).

MISSION-CJ in DTCs is delivered by a case manager and peer support specialist team for up to 12 months. The MISSION-CJ curriculum includes a facilitator manual, consumer workbook, and fidelity tool to support model implementation [15]. MISSION-CJ fidelity is monitored and supported through initial and refresher training via MISSION U, a standardized interactive web-based facilitator training curriculum; use of the MISSION-CJ fidelity tool, a standardized weekly fidelity tracking form which tracks service delivery and engagement with MISSION-CJ components, monthly fidelity case conferences, and quarterly fidelity data reviews.

Randomized controlled trials of MISSION [19] have demonstrated a positive impact on behavioral health and other outcomes. Two MISSION versus treatment-as-usual (TAU) studies demonstrated improved treatment attendance, outpatient treatment engagement, reduced re-hospitalization, and improved mental health and SUD outcomes. Several pre-post, within-subject MISSION-CJ pilot studies have documented positive outcomes [20–23]. Specific to DTCs, one MISSION-CJ pilot study in a suburban DTC (*N* = 86) found: 1) significant reductions in incarceration (*p* < .01) and alcohol/drug use (*p* < .01); 2) improved mental health symptoms on the Behavior and Symptom Identification Scale (BASIS) total score (*p* < .043), relation to self and others (*p* < .02), impulsive/addictive behaviors (*p* < .001), and depression and functioning subscales (*p* < .033); 3) reductions in hospitalizations (*p* < .01); and 4) increased employment (*p* < .01) at 6-month post-enrollment [24]. Another MISSION-CJ pilot study in a rural DTC (*n* = 57) found that at 6-month post-enrollment, participants reported: 1) reduced incarceration (*p* < .001), arrests (*p* < .001), and illicit drug use (*p* < .046); 2) reduced mental health symptoms (*p* < .031) and trauma symptoms (*p* < .008); and 3) increased employment (*p* < .05). This study also found high rates of treatment/support linkage and engagement, with 76% of participants receiving medication-assisted treatment and 75% attending self-help groups at discharge [20].

Despite MISSION-CJ improving treatment outcomes for those with a COD in DTCs [20,25–28], racial/ethnic disparities in treatment engagement and outcomes persist. Twelve-month outcome data indicated that Black/African American and/or Hispanic/Latino MISSION-CJ participants: 1) experienced a twofold increase in nights spent in jail in the past 6 months; 2) were four times as likely to report injection drug use in the past 6 months; and 3) experienced 25% fewer behavioral health improvements relative to their White counterparts. Furthermore, Black/African American and/or Hispanic/Latino participants were 38% less likely to graduate from MISSION-CJ [26].

A focus group with MISSION-CJ participants identified the following engagement barriers for Hispanic/Latino participants: long wait time for interpreters, lack of continued recovery supports post-graduation, and lack of health insurance [29]. However, this was only one focus group in one court, did not include Black/African American participants, and did not use a rigorous qualitative methodology with a health equity implementation lens. To date, limited attention has been paid to racial/ethnic COD treatment disparities in criminal legal settings, especially framing these disparities as a special case of implementation failure [30] where enhanced community- and provider-informed strategies are needed to simultaneously enhance uptake and advance equity [31].

### Equitable Implementation of Evidence-Based Programs in Criminal Legal Settings

Despite growing attention on developing and implementing EBPs for adults with behavioral health conditions in criminal legal settings, EBPs have not been implemented equitably [32]. Equitable implementation occurs when strong equity components – including explicit attention to the culture, history, values, assets, and needs of the community – are integrated into implementation of EBPs [33].

Equitable implementation of EBPs in criminal legal settings is not well understood. Traditionally, EBP implementation in criminal legal settings has occurred without integrating equity considerations and components, nor inviting people with lived experience (participants and providers) to participate in the development of and decision-making about the intervention and its implementation. These omissions have contributed to inequitable implementation and require improved engagement with diverse community and staff/providers to inform future efforts [32].

**Fig. 1.**
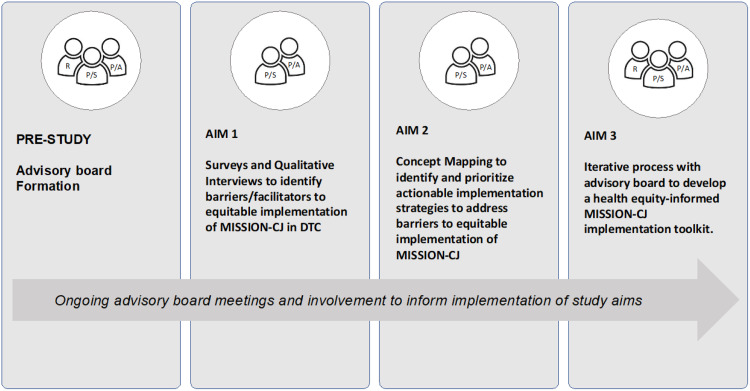
Graphical representation of the study protocol: a community-engaged protocol to gather and utilize lived experiences from MISSION-CJ/Drug Court participants and related providers and administrators, to create a multilevel understanding of barriers and facilitators to equitable implementation of MISSION-CJ and create a health equity informed implementation toolkit to help increase equitable access and engagement in MISSION-CJ. P/S=Person Served, P/A=Providers/Administrators, R = Researchers.

Few studies in criminal legal settings have used health equity-informed approaches to systematically identify, select, and/or evaluate implementation strategies, defined as methods or techniques used to enhance the adoption, implementation, and sustainability of an EBP or practice [34]. We address this gap via the following three specific aims (see Fig. 1 for a graphical representation of the study):

Aim 1: Identify facilitators and barriers to equitable implementation of MISSION-CJ via surveys and qualitative interviews with 30 Black/African American and/or Hispanic/Latino persons served by MISSION-CJ in DTCs and related providers/administrators.

Aim 2: Identify and prioritize actionable implementation strategies to address barriers to equitable MISSION-CJ implementation in DTCs using concept mapping with 60 Black/African American and/or Hispanic/Latino persons served by MISSION-CJ in DTCs and related providers/administrators.

Aim 3: Develop a health equity-informed MISSION-CJ implementation toolkit via an iterative development process with our advisory board.

This study will bridge this gap by bringing together implementation science, health disparities, and community-engaged approaches to understand and address drivers of racial/ethnic behavioral health treatment disparities among adults in DTCs.

## Methods

This mixed-methods study protocol will identify multilevel drivers of MISSION-CJ implementation disparities among Black/African American and Hispanic/Latino individuals with COD in DTCs. To identify these drivers, we will utilize a QUAN + QUAL mixed-methods approach [37], where we will simultaneously collect and analyze quantitative and qualitative data, giving equal weight to both. Next, we will match barriers and facilitators to implementation strategies with an advisory board to enhance equitable implementation of MISSION-CJ within DTCs. This study will be conducted in collaboration with persons served, providers, and administrations in DTC and was approved by our university’s Institutional Review Board. See Table 1 for study methods.

**Table 1.** Study methods

Methods	Aim 1	Aim 2	Aim 3
**Aim**	Identify facilitators and barriers to equitable implementation of MISSION-CJ	Identify and prioritize actionable implementation strategies to address barriers to equitable implementation of MISSION-CJ	Develop a health equity-informed MISSION-CJ implementation toolkit
**Participant eligibility criteria**	Persons served (*n* = 15): adults (18+), self-identify as Black/African American and/or Hispanic/Latino, Have ever received MISSION-CJ (this includes current participants, graduates and non-completers) within four geographically diverse DTCs in one New England state. Providers/Administrators (*n* = 15): Adults (18+) Have ever provided treatment, judicial, and wraparound support services to persons served within the MISSION-CJ programs in the four DTC study sites. Providers can include judicial (e.g., judge, probation officer, specialty court leadership), treatment (e.g., MISSION-CJ case manager, peer support specialist, community-based trauma counselor), and community/other (e.g., interpreter, employment coach) providers	1. Persons served (*n* = 30): Same as Aim 1 2. Providers/Administrators (*n* = 30): Same as Aim 1	Persons served: (*n* = 6) Same as Aim 1 Providers/Administrators (onstrated expertise in behavioral health, equity, community engagement, MISSION-CJ and/or the criminal legal system
**Data source**	Persons served, providers, and administrators	Persons served, providers, and administrators	Persons served, providers, and administrators
**Data source level** **measurements**	Individual/self	Individual/self	Individual/self
**Outcome/** **deliverable**	List of identified barriers/facilitators	Preliminary list of community generated implementation strategies to advance equity rated by feasibility and importance	Toolkit

### Guiding Implementation Framework

**Fig. 2.**
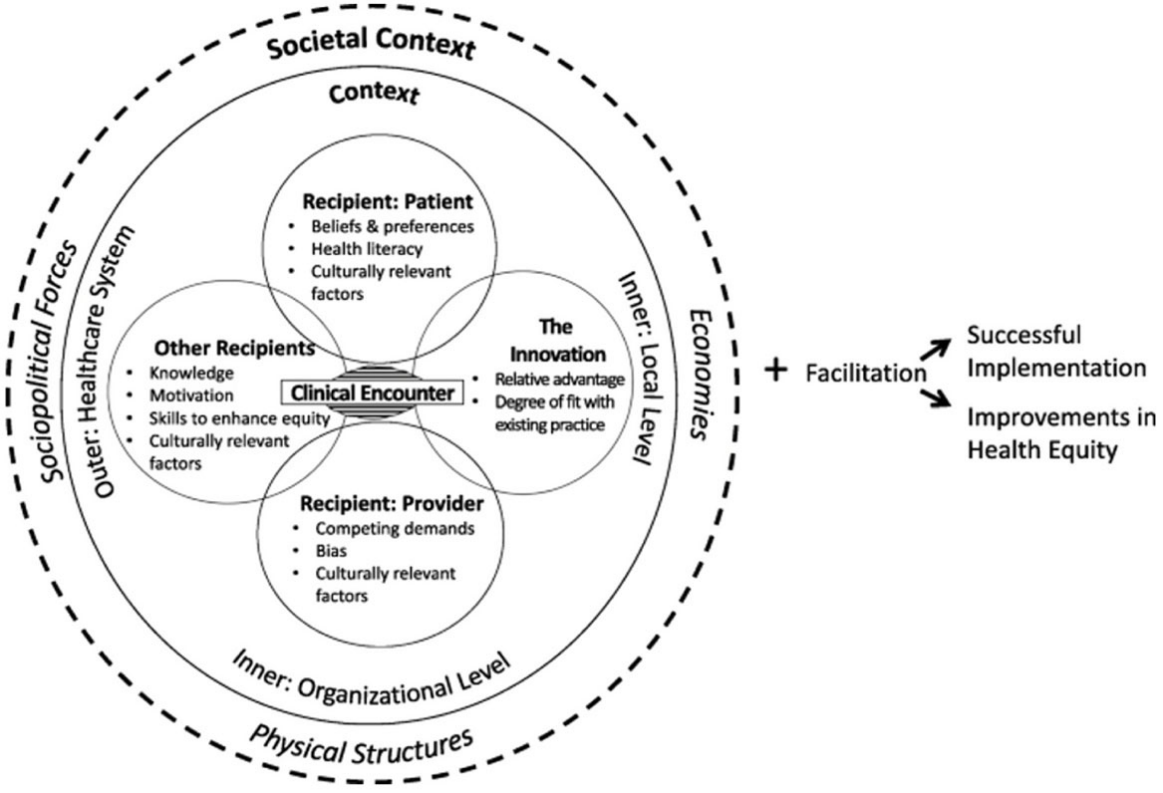
Health equity implementation framework, study guiding framework. *Figure reproduced with permission from Woodward, et al. (2019)* ^35^.

This study is guided by the Health Equity Implementation Framework (HEIF) (see Fig. 2), an implementation science determinant framework specifically designed to inform equitable implementation. The framework proposes multiple levels of implementation determinants (i.e., systems level, recipients, and characteristics of the intervention), including equity determinants (i.e., drivers of disparities, such as culturally relevant factors of recipients and providers, societal context including structural racism in policies). The HEIF also suggests the most effective implementation strategies must be multifaceted to account for these distinct and multiple levels [35,36].

### Advisory Board

Study processes will be informed by an advisory board formed prior to study commencement. The board will consist of two Black/African American and/or Hispanic/Latino people with lived experience with MISSION-CJ and/or DTC, two MISSION-CJ and/or DTC providers/administrators, and two researchers, the study PI and HEIF developer. The advisory board was instrumental in developing the research question and will inform study design, instrument development, and recruitment. Our board members will generate a list of 5–6 additional potential advisory board members, representing persons served by MISSION-CJ in DTC, persons with lived experience of COD and criminal legal involvement, providers, and/or administrators. All board members will be compensated for their time and meet monthly for 90–120 minutes.

Given the significant power imbalances and histories of oppression between people of color impacted by the criminal legal system, providers within this systems, and researchers, we will consult literature [38] and our advisory board members for best practices for inclusive and ways to engage persons served as community partners in the advisory board, elevate community voices, and ensure power sharing. A separate advisory board or separate initial advisory board meetings with persons served may be more appropriate to work through their concerns (e.g., fear of voicing concerns with judicial providers present) and build trust. We will review best practices during board meetings and through anonymous polling; the board will select the final set of processes (see Table 2 for advisory board processes).

**Table 2.** Advisory board proposed processes. Adapted from Newman SD, Andrews JO, Magwood GS, Jenkins C, Cox MJ, Williamson DC. Community advisory boards in community-based participatory research: a synthesis of best processes. Prev Chronic Dis 2011; 8(3): A70.

Processes	Task	Proposal
**Formation**	Clarify purpose	Integrate community perceptions, preferences, and priorities in the development of a research agenda and processes. Advise on study protocol design and implementation. Evaluate MISSION-CJ materials and implementation. Provide feedback on study recruitment plan.
	Clarify functions/roles	Members will serve as partners and will bring issues and concerns from the community to the table. Through board discussion, these issues will be resolved in a manner that is mutually beneficial to both the research team and the community (e.g., integrated into the research design, flagged for future research, etc.).
	Determining membership composition and recruitment strategies	Initial advisory board will brainstorm ways to identify potential additional CAB members and determine the best recruitment and selection strategies via an iterative process. Create and utilize a "potential member matrix" that includes people to be considered; initial advisory board inclusion criteria, and specific items to assess their fit with the intent and purpose of the board.
	Commitment documentation	Utilize a document outlining the roles and scope of work for each board member. Document will be signed by both the board member and PI.
**Operation**	Operating procedures	Written procedures focused on group dynamics, including how to support board members in listening to one another, demonstrate mutual respect, manage disagreements, etc. (captured in group agreements or rules) Members will periodically reassess and revise the procedures.
	Operating principles	Define community values or principles that guide research (e.g., good and accessible care should be available for all). Members will continually come back to these principles to assess if advisory board focus, interaction, processes, and decisions are aligned with these principles.
	Establishing leadership, balancing power, and making decisions	Select a community member as an advisory board co-chair. Use democratic and consensus-based decision-making (e.g., individual anonymous polling). Establish a protocol for decision-making (e.g., use the 70% majority, a commonly used cutoff for consensus in community advisory boards) Utilize subcommittees to decentralize decision-making, balance power, and match individual traits (e.g., some members may talk more freely in smaller dyads or groups).
**Evaluation**	Evaluating processes	Periodic anonymous surveys soliciting feedback on board processes and experiences. Continual monitoring of process data (e.g., member turnover).
**Sustainability**	Sustainability	Ask board to define of sustainability and the criteria to evaluate board sustainability. Recognize membersʼ contributions (e.g., compensation $50 per meeting, welcome basket, board certificate, virtual kick off pizza party).

Our culturally diverse and multidisciplinary study team includes at least one team member who is bilingual and at least one team member with lived experience. Initial and ongoing study training will focus on community-based participatory research, health equity, social justice, implementation science, and cultural and structural humility.

## Study Aims & Method

Aim 1: Identify Facilitators and Barriers to Equitable Implementation of MISSION-CJ Using a Mixed-Methods Approach via Surveys and Qualitative Interviews with 30 Black/African American, and/or Hispanic/Latino Persons Served by MISSION-CJ in DTCs and Related Providers/Administrators.

Participants: Fifteen persons-served and 15 providers/administrators will be invited to participate in an individual qualitative interview and complete a brief survey. See Table 1 for eligibility criteria.

Procedures: Participants will be recruited from four geographically diverse DTCs with MISSION-CJ program in one New England state. Persons served will be informed about the study via brief verbal announcements made by the research team during MISSION-CJ programming in English and Spanish. Interested individuals will be invited to a brief phone or video conference screening conducted to determine eligibility, review study procedures, and conduct the process of informed consent. All study team members will be trained to maintain confidentiality of participants. During recruitment and study procedures, we will emphasize that participation is not shared with the court, does not affect their receipt of services, and their responses are de-identified.

The following approaches will be used to minimize common barriers to racial/ethnic minoritized groups’ research participation [39], including providing several opportunities to learn and ask questions about the research process; closely reviewing human subject protection measures to address fears of exploitation; explicitly addressing concerns about unintended consequences; clarifying confidentiality procedures; minimizing perceptions of coercion by not recruiting persons served during or as part of the court session and clearly outlining how participation will not impact court standing or criminal legal cases; and utilizing a diverse and bilingual study team. Feedback from individuals with lived experience will inform wording and materials used in this process.

Providers will be informed about the opportunity to participate via brief verbal announcements made by the study team at pre-court staff meetings, MISSION-CJ team meetings, and relevant state-wide provider meetings and trainings. Interested providers will be invited to a brief screening conducted by the study coordinator to determine eligibility, review study procedures, and review the informed consent process.

All eligible individuals will be scheduled for an individual interview in the language of their choice and sent a brief survey. Interviews will last 45–60 minutes via phone or Zoom and audio-recorded [40,41]. Depending on COVID-19 restrictions, the interviews could be offered in person, by phone, or virtually. At the scheduled interview time, the study reviewer will thoroughly review the study purpose and confidentiality again. At the end of the interview, participants will be compensated with a $50 gift card. If participants decide to not be audio-recorded at any point during the interview, notes will be taken, and interviewees will be provided a copy of interviewer notes for the purposes of verifying accuracy, correcting errors or inaccuracies, and providing clarifications. Combined with careful interviewer training and consistent supervision, this process can effectively capture interview content [40,41]. Audio-recorded interviews will be transcribed verbatim, de-identified, and reviewed for transcription accuracy.

## Measures

### Survey

***Persons Served.*** The survey will gather demographic information including age, gender, race/ethnicity, sexual orientation, educational level, language, immigration history/status, family characteristics (e.g., number of children), housing status, income, and religion and questions about their pathway to, and tenure in, MISSION-CJ and DTC (e.g., Who referred you?, How long have you been in the program?). Their perceptions of MISSION-CJ and DTC providers’ cultural competency will be measured by an adapted version of the Iowa Cultural Understanding Assessment–Client Form [42]. The Iowa Cultural Understanding Assessment–Client Form scale is a brief assessment for understanding organizational culture through the lens of client perspectives based on staff–client relations, environmental factors, and service delivery processes. This measure was selected because it quantitatively assesses aspects of HEIF’s three health equity domains which are important in understanding equitable implementation. The survey, offered in English and Spanish, will take 10–15 minutes, and can be completed online, by phone, or by pen/paper. The survey was written at a 6th grade reading level. Participants can opt to have the survey read to them by the interviewer and verbally respond to survey questions.

***Providers**.* The survey will provide information about the providers’ demographics including: gender, race/ethnicity, and educational level; organizational cultural competence assessment; and self-assessment of their own cultural competency. The organizational and self-assessment questions were adapted from the work of the Association of University Centers on Disabilities (AUCD) Multicultural Council’s Assessment of Organizational Cultural Competence, which [43] assists organizations in assessing their progress towards cultural competence, both at the organizational and individual level. The survey will take about 10–15 minutes to complete and can be completed online, by phone, or pen/paper.

### Interview

We will conduct semi-structured individual interviews with participants to identify perceived barriers and facilitators to MISSION-CJ implementation in Drug Court. The interview guide will be based on HEIF components and will specifically elicit perspectives on the following topics: needs of both persons served and providers/administrators in regard to COD treatment and its delivery for the target populations; current implementation of MISSION-CJ and DTC; multilevel factors that might facilitate or inhibit equitable implementation of MISSION; and ideas about how to address identified barriers. HEIF-informed semi-structured interview guides (see supplemental materials for sample interview questions by HEIF domain) will be developed using iterative community and provider feedback. Iterative drafts of the interview guide will be edited by the advisory board, study team, and an external qualitative researcher. Thirty interviews will be completed – 15 per group or until the data collected reaches saturation. Sample sizes of 10–15 [44] per group have been found to meet the threshold for data saturation in qualitative research [45].

Analytic Plan: Once data are collected, cleaned, and entered in respective software for management (SPSS for quantitative data from surveys, Dedoose for qualitative data from semi-structured interviews), each data type (qualitative data from interviews and quantitative data from surveys) will be analyzed independently and then merged and presented to the study team to integrate quantitative and qualitative findings. Directed content analysis [46] will be used for qualitative data to categorize according to domains from the HEIF. Coding will be completed by the study team – the PI (coder A), two doctoral level coders (coders B and C), and one bachelor’s in psychology level coder (coder D). Coders will extract data on specific concepts related to implementation, disparities, culture, cultural identities, and equity (see Table 3 for coding definitions) and barriers or facilitators of MISSION-CJ implementation. Quantitative survey data will be analyzed, and descriptive statistics will be generated. Mean responses to survey items will be compared by groups (e.g., drug court sites, provider roles, gender, race, ethnicity, etc.) to determine differences between specific groups (e.g., persons served ratings on program cultural competency vs. providers/administrators’ ratings of program cultural competency).

**Table 3.** Coding definition

Coding	Coding Definition
Implementation	A specified set of activities, methods, and strategies designed and used to put into practice a practices or program.
Disparity	Experiences, perceptions, or reasons for unjust differences in treatment.
Culture	Shared characteristics of a group of people that structures the way people view the world. Set of beliefs, norms, and values that influence ideas about the nature of relationships, the way people live their lives, and the way people organize their world.
Cultural identities	Includes age, developmental disabilities, acquired disabilities, religion, ethnicity, sexual orientation, socioeconomic status, indigenous group membership, nationality, gender, gender identity, and gender expression, and veteran status. This broad definition allows for exploration of multiple intersecting cultural identities.
Equity	Equity is an ongoing process of assessing needs, acknowledging and correcting historical inequities, addressing social determinants (e.g., employment and housing stability, insurance status, proximity to services, and culturally responsive care), and creating conditions for optimal outcomes by members of all cultural identity groups.

The qualitative data will be quantified. For example, presenting ever-coded (e.g., the number of transcripts that had the code assigned ever) and frequency (e.g., the number of times the code was assigned throughout all transcripts) counts will provide additional data to support salience of emergent themes. We will use a joint display to merge the two data types by illustratively bringing them together to complement understanding and interpretation by detailing codes, themes, and survey responses to engage in a side-by-side comparison. This strategy has been used in prior studies to identify points of convergence and divergence in participant responses [47] and provides a more complete, unified explanation than either single data type alone.

### Aim 2: Identify and Prioritize Actionable Implementation Strategies to Address Barriers to Equitable MISSION-CJ Implementation in DTCs Using Concept Mapping with 60 Black/African American and/or Hispanic/Latino Persons Served by MISSION-CJ in DTCs and Related Providers/Administrators

#### Methods/Design

Participants: Thirty persons served and 30 providers/administrators will be invited to participate in a concept mapping activity. Persons served and provider/administer eligibility criteria will be the same as outlined in Aim 1.

Procedures: Concept mapping is a mixed-methods approach to engage community members and providers in the community-based participatory research process [48]. Concept mapping has been effectively used to bring together diverse groups of community members and providers to expeditiously create an interpretable conceptual framework that can serve as a foundation for implementation planning. Concept mapping generates group consensus on key ideas and supports systems thinking by eliciting individual experiences to identify group conceptualization of an issue. It equalizes or lessens power differentials among participants by giving individuals equal voice and choice and engages persons served and providers as both study participants and collaborative decision-makers. Concept mapping tasks such as sorting and ranking (described below) are done individually and anonymously, so everyone gets an anonymous vote on the final conceptual naming and grouping of clusters. Since statistical procedures analyze the sorting and rating data, people do not need to discuss, defend, or develop consensus with other participants and no one person holds the decision-making power.

The concept mapping process has qualitative and quantitative components that can be conducted online, face to face (via phone or video conferencing), or in combination. The qualitative elements are ***brainstorming ideas*** from a prepared focus question, ***structuring the ideas*** by sorting them into thematic groups based on their perceived similarity and then rating them on importance and feasibility, and ***analyzing and interpreting*** the concept maps generated. The quantitative elements are rating the qualitative statements generated and creating different visual maps to convey key concepts. Typically, concept mapping is organized into four structured and sequenced activities: (1) brainstorming, (2) sorting, (3) rating, and (4) data interpretation.

It is important to note that this sequence does have the potential for participant burden [49]. Therefore, the concept mapping sequence in this study will be modified such that statements will be extracted from our Aim 1 qualitative interviews; sorting and rating will follow the typical concept mapping sequence; and interpretation of the concept maps will be done by an advisory board. We considered traditional concept mapping, but due to concerns of participant burden, we opted to use this adapted version which has been successfully tested by others [50]. The traditional concept mapping methodology comprises generation of ideas (statements/items) through focus group brainstorming guided by a study-specific prompt or focus question. Instead, the generation of ideas will be synthesized from the Aim 1 individual qualitative interviews. The interviews will include prompts or focus questions such as “List key strategies to ensure MISSION-CJ is reaching and benefitting all people in Drug Court”; “To eliminate disparities and achieve health equity in the treatment of COD within Drug Courts, programs should…”; “Thinking as broadly as possible, what are some strategies to ensure MISSION-CJ is reaching and benefitting people of color within the Drug Court?; and “What are the most impactful and feasible strategies to improve MISSION-CJ services for Black/African American and/or Hispanic/Latino people in Drug Court?” We will fine-tune these questions with our advisory board and pilot test the question with at least one person served and one provider.

Study participants will be asked to identify and prioritize actionable implementation activities to address barriers to equitable MISSION-CJ implementation in DTC identified in Aim 1. Concept mapping is usually an individual virtual activity. To account for technology access, literacy, and comfortability, participants will be given the options to complete the activity virtually, in-person, or by video conference. If participants are interested in completing the activity virtually, they will be provided with computer access and support as needed. The same recruitment and screening strategy described in Aim 1 will be utilized, but the study will be expanded to additional DTCs to capture additional diversity of community and provider perspectives across different DTCs in the state. Our planned sample size is slightly higher than the recommended sample size for concept mapping (*n* ≥ 15 per group) [51] to ensure we can make meaningful comparisons between persons served versus providers/administrators.

Those who agree to participate virtually will be sent a link to the online platform in either English or Spanish. The site will be available to participants for a 1-month period. On the site, participants will provide brief demographic information. Concept mapping will include a sequential process with the following steps. First, participants will view and read a list of implementation strategies synthesized from Aim 1. For the sorting task, participants will be instructed to sort the strategy statements into categories that make sense to them and to generate a label for each category they created. Next, for the rating task, all participants will be asked to rate the importance of each strategy statement on a scale ranging from 0 (not important at all) to 5 (extremely important) based on the impact each strategy would have on the equitable implementation of MISSION-CJ. All participants will then be asked to rate each strategy statement on its feasibility using the scale 0 (not feasible at all) to 5 (extremely feasible).

If the participant elects to complete concept mapping in-person, an interviewer will meet with them and guide them through the concept mapping activity on a study laptop in person. The interviewer will walk them through the steps outlined above to elicit and record participant responses in the Concept System® groupwisdom™ platform. Upon completion, participants will receive a $50 gift card. Concept mapping has been conducted successfully with similar participant populations [52].

Analyses Plan: The Concept System® groupwisdom™ [53] will be used to conduct the analyses. The software uses multidimensional scaling and hierarchical cluster analysis to interpret sorting and rating data and to produce visual representations of the data (e.g., maps). Hierarchical cluster analysis, using input from multidimensional scaling, mathematically groups each statement into adjustable cluster solutions, based on how participants rated and sorted the data. Each cluster represents a unique theme on the resulting map. Descriptive statistics for the importance and feasibility ratings will be calculated. Each implementation strategy’s importance and feasibility score will be plotted on a graph. The PI will present results to the advisory board to understand and interpret concept mapping results.

### Aim 3: Develop a Health Equity-Informed MISSION-CJ Implementation Toolkit via an Iterative Development Process with Our Advisory Board

#### Methods/Design

Procedures: Data summaries from Aims 1 and 2 will be reviewed with our advisory board who will help interpret the findings to inform development of a toolkit to advance equitable implementation of MISSION-CJ in DTCs. The PI will facilitate discussion and gather consensus for modifications.

Toolkit Development: The study team will draft and refine toolkit content with iterative feedback from the advisory board. Each of the newly developed components will be beta-tested with the board and their feedback will inform the final version of the toolkit to be used in a larger trial. The board will provide commentary on what they like, dislike, and changes for improvement. For example, if implementation strategies such as “strategies to increase ongoing person served feedback on the MISSION-CJ implementation; identifying/preparing a provider and/or person served program champion, or culturally tailoring MISSION-CJ components” are suggested as important and feasible strategies to advance equitable implementation in Aims 1 and 2, the advisory board will review toolkit sections related to these strategies (e.g., sample focus group instructions and questions to be used by programs to solicit participant/family feedback). Utilizing the nominal group technique, we will present a toolkit section (e.g., strategies to identify and prepare graduates of color to serve as program champions) then (1) ask members to silently generate ideas about how best to enhance it, (2) gather round-robin feedback from group members to record each idea, (3) discuss each recorded idea for clarification and evaluation, and then (4) conduct individual and anonymous polling to identify priorities for further refinement. The final toolkit will be assessed by board members via the Acceptability of Intervention Measure, Intervention Appropriateness Measure, and Feasibility of Intervention Measure [55], four-item measures to determine to what extent the toolkit is acceptable (e.g., Is it agreeable, palatable, satisfactory?), appropriate (e.g. Is it relevant or a fit?), and feasible (e.g., Can it be successfully used in DTCs?).

The toolkit will help MISSION-CJ end users (providers, courts, and systems) use strategies to close gaps in behavioral healthcare outcomes that result from multilevel and inequitable policies and practices. The toolkit will include:

- Supporting evidence on why both uptake and equity are important;
- Tactical and practical steps to equitably implement evidence-based COD programming in DTC;
- Strategies to address issues arising from variation and obstacles within settings;
- Strategies to evaluate and enhance equitable implementation of evidence-based COD programming in DTC;
- Suggested training to ensure staff and leadership share a common understanding of the complexities of inequities and have the skills to advance health equity.

## Discussion

There is an urgent need for research to inform and address multilevel drivers of health disparities in criminal legal settings and populations. To our knowledge, this is one of the first studies to utilize a community- and provider-informed approach to explore racial/ethnic behavioral health treatment disparities in DTCs. The exploratory and developmental work proposed in this study will address research gaps and provide actionable implementation guidance to advance equitable implementation of COD interventions in DTCs.

We hypothesize that a community and provider co-developed toolkit will likely contribute to enhanced equitable implementation of COD interventions within DTCs. Given the novel and pragmatic nature of the study, we envision this work will have a positive public health impact on the lives of adults with COD receiving care as part of their criminal legal involvement. This work can provide valuable insights on the practical aspects of conducting research within criminal legal settings and populations with criminal legal involvement, especially community-engaged research. Lastly, this study provides an example of the application of Dissemination and Implementation Science to advance translational research in a criminal legal setting.

## Supplementary material

For supplementary material accompanying this paper visit https://doi.org/10.1017/cts.2023.14. click here to view supplementary material
