# Supplementary material for: Identifying strategies to advance equitable implementation of co-occurring mental health and substance use disorder treatment in drug treatment courts: A study protocol
Source: J Clin Transl Sci. 2023 Feb 7;7(1):e80. doi: 10.1017/cts.2023.14 (PMC10130840; doi:10.1017/cts.2023.14)
Supplement: Supplementary file 1 [file S2059866123000146sup001.docx]

| **HEIF Domains**  Supplemental Table 1: Sample Interview Guide Questions by HEIF Domain** | **Definition** | **Person Served Sample**  **Questions** | **Provider/Administrator Sample Questions** |
| --- | --- | --- | --- |
| Characteristics of the Innovation | Characteristics related to the intervention (e.g., MISSION-CJ) itself, such as its usability (e.g., unintended negative consequences of participation, modes of delivery), its relative advantage over existing treatments, or its trialability for patient. | *What parts of MISSION-CJ work well for you?*  *What parts of MISSION-CJ do not work well for you?* | *What are some strengths of MISSION-CJ?*  *What components of MISSION-CJ would you change to better serve Black/African American, and/or Hispanic/ Latino individuals in the program?* |
| Clinical Encounter (Patient-Provider Interaction) | The moment when the patient is exposed to or offered the innovation by a provider or staff. The interaction between characteristics of the innovation and recipients (e.g., person served and MISSION-CJ case manager, person served and DTC clinician, etc.). | *Tell me about your interactions with MISSION-CJ providers.*  *How did they describe MISSION-CJ to you?* | *Tell me about your interactions with persons served in MISSION-CJ.*  *What types of cultural* considerations come up in your interactions with persons served?* |
| Recipients: Culturally Relevant Patient Factors | Person receiving or being offered the innovation. Cultural factors include demographic factors, beliefs, information, and also biological, psychological, or genetic conditions related to equitable implementation. | *Tell me about your cultural background.*  *What aspects about your cultural background would you like your providers to consider in your care?*  *You mentioned that you identify as [client’s self-described cultural* identities], how does your MISSION team consider your cultural background in care?* | *Tell me about the cultural backgrounds of people you serve in the program.* |
| Recipients: Culturally Relevant Provider Factors | Providers’ objectives and beliefs about a patient, health problem, or innovation, that affects how they behave during the patient-provider interaction, including bias, both implicit and explicit, and demographics. | *Tell me about your MISSION Case Manager and Peer Support Specialist (PROBE about provider background, perceived beliefs about population, COD, MISSION).* | *Tell me about your cultural background.*  *How do you assess persons served cultural identities, beliefs, needs, and strengths?* |
| Other Recipients: Culturally relevant factors | Other recipients include individuals who affect the delivery of an innovation, such as a court administrator or clerk. Their objectives and beliefs about a patient, health problem, or innovation, that affects how they behave during interactions with patients, including bias, both implicit and explicit, and demographics. | *Tell me about any other individuals who might impact your experience in MISSION-CJ, such as a court administrator or clerk, court security personnel, interpreters, etc.*  *How have they treated you?* | *Tell me about any other individuals who might impact the delivery of MISSION-CJ, such as a court administrator or clerk, court security personnel, interpreters, etc.*  *Is there anything they do that hinders or supports people receiving MISSION-CJ?* |
| Inner Local Level & Organizational Context | Inner context factors at the local or organizational level can include leadership support for an innovation, feedback processes, the structure of a system, or any formal policies to support or hinder equitable implementation. | *What makes it challenging or more difficult to participate in drug court?*  *What would you change about drug court?* | *What are some barriers to participating in Drug Treatment Court (DTC)?*  *What are some barriers to implementing MISSION-CJ in DTCs?*  *What are some things in the wider court system that might impact care for Black/African American and/or Hispanic/Latino adults with co-occurring mental health and substance use disorder and criminal legal involvement?*  *What can be done to improve access, engagement, and outcomes for this population in drug court?* |
| Outer Context: Health Care System | The macro-level of outer context includes the wider healthcare system and effect this has on the other domains. In this study, the outer context is the Criminal Legal System.  We also propose to ask about the Behavioral Health Treatment System. | *How have you been treated in the criminal legal system?*  *(PROBE for experiences of prejudice, discrimination, and stereotyping)*  *How has this treatment impacted your experience in MISSION-CJ? DTC?*  *How have you been treated in the systems that provide treatment for mental health and/or substance use? (PROBE for experiences of prejudice, discrimination, and stereotyping)*  *How has this treatment impacted your experience in MISSION-CJ? DTC?* | *How have people’s experiences in the wider criminal legal system impacted their experience in MISSION-CJ? DTC?*  *How have people’s experiences in the behavioral health treatment system impacted their experience in MISSION-CJ? DTC?* |
| Societal Influence: Factors outside of the system, that may impact the healthcare delivery--society at large, macrosystem-level influences, structures of linked institutions on equitable implementation. | | | |
| Economies | How are goods and services exchanged in health care, how care is obtained financially or affected by exchange of services, including insurance, patient income, etc. | *What are some financial factors that impact your access to and participation in MISSION? Financial factors can include things like money, benefits, insurance, costs like bus fare, things like that.* | *What are some economic barriers and/or incentives to participating in MISSION for persons served?*  *What some economic barriers and/or incentives to implementing MISSION in DTC?* |
| Physical Structure | The physical environment, or the built environment's impact on equitable implementation. | *What are some things in the physical environment that help you access and participate in MISSION-CJ?*  *What are some things in the physical environment that make it hard for you to access and participate in MISSION-CJ?* | *What are some barriers in the physical environment that impact access and engagement in MISSION-CJ services?* |
| Sociopolitical forces | Policies and procedures, informal or formal, in our national and local governments that systemically inhibit or promote equitable health or equitable implementation. | *What are some things in our larger society that might impact your care?*  *Probe for policies, procedures, laws, media attention, and things going on in the community.* | *What are some things in our larger society that might impact care for Black/African American, Hispanic, and/or Latino adults with co-occurring mental health and substance use disorder and criminal legal involvement?* |
| * Note: Cultural identities are defined broadly to include age, developmental disabilities, acquired disabilities, religion, ethnicity, sexual orientation, socioeconomic status, indigenous group membership, nationality, gender, gender identity, and gender expression, and veteran status, allowing for exploration of intersecting cultural identities.  ** Questions will be refined via iterative feedback from the Advisory Board. | | | |

*Adapted with permission from: Woodward et. al., 2021.*
